# Dopaminergic modulation of olfactory‐evoked motor output in sea lampreys (*Petromyzon marinus* L.)

**DOI:** 10.1002/cne.24743

**Published:** 2019-07-24

**Authors:** Philippe‐Antoine Beauséjour, François Auclair, Gheylen Daghfous, Catherine Ngovandan, Danielle Veilleux, Barbara Zielinski, Réjean Dubuc

**Affiliations:** ^1^ Département de neurosciences Université de Montréal Montréal Québec Canada; ^2^ Département des sciences de l'activité physique Université du Québec à Montréal Montréal Québec Canada; ^3^ Department of Biological Sciences University of Windsor Windsor Ontario Canada

**Keywords:** anatomical tracing, dopamine, immunofluorescence, lamprey, locomotion, olfaction, olfactory bulb, RRID: AB_2314665, RRID: AB_2336881, RRID: AB_2338881, RRID: AB_2534079, RRID: AB_390204, RRID: AB_94817

## Abstract

Detection of chemical cues is important to guide locomotion in association with feeding and sexual behavior. Two neural pathways responsible for odor‐evoked locomotion have been characterized in the sea lamprey (*Petromyzon marinus* L.), a basal vertebrate. There is a medial pathway originating in the medial olfactory bulb (OB) and a lateral pathway originating from the rest of the OB. These olfactomotor pathways are present throughout the life cycle of lampreys, but olfactory‐driven behaviors differ according to the developmental stage. Among possible mechanisms, dopaminergic (DA) modulation in the OB might explain the behavioral changes. Here, we examined DA modulation of olfactory transmission in lampreys. Immunofluorescence against DA revealed immunoreactivity in the OB that was denser in the medial part (medOB), where processes were observed close to primary olfactory afferents and projection neurons. Dopaminergic neurons labeled by tracer injections in the medOB were located in the OB, the posterior tuberculum, and the dorsal hypothalamic nucleus, suggesting the presence of both intrinsic and extrinsic DA innervation. Electrical stimulation of the olfactory nerve in an in vitro whole‐brain preparation elicited synaptic responses in reticulospinal cells that were modulated by DA. Local injection of DA agonists in the medOB decreased the reticulospinal cell responses whereas the D2 receptor antagonist raclopride increased the response amplitude. These observations suggest that DA in the medOB could modulate odor‐evoked locomotion. Altogether, these results show the presence of a DA innervation within the medOB that may play a role in modulating olfactory inputs to the motor command system of lampreys.

## Graphical abstract

Dopaminergic innervation in the medial olfactory bulb was shown to modulate olfactory inputs to the motor command system. Dopaminergic processes were anatomically traced back to local and diencephalic DA neurons. Furthermore, microinjection of DA agonists in the medOB decreased reticulospinal cell responses to olfactory stimulation.

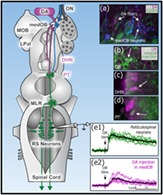

**Abbreviations**
BSA: bovine serum albumin
DA: dopamine
DHN: dorsal hypothalamic nucleus
EPSPs: excitatory postsynaptic potentials
G: glutaraldehyde
GSIB4: *Griffonia simplicifolia* isolectin B4
LPal: lateral pallium
medOB: medial olfactory bulb
MLR: mesencephalic locomotor region
MOB: main olfactory bulb
MRRN: middle rhombencephalic reticular nucleus
OB: olfactory bulb
ON: olfactory nerve
PBS: phosphate‐buffered saline
PT: posterior tuberculum
RS: reticulospinal
SNc: substantia nigra, pars compacta
TBS‐m: Tris 0.05 M with 1.0% sodium metabisulfite
TH: tyrosine hydroxylase
VTA: ventral tegmental area

## 1 INTRODUCTION

Lampreys represent the oldest extant group of vertebrates and their behavior is strongly influenced by olfactory inputs. Sea lampreys (*Petromyzon marinus* L.) rely heavily on the detection of chemical cues for feeding (Kleerekoper & Mogensen, 1963) and sexual behaviors (Buchinger, Siefkes, Zielinski, Brant, & Li, 2015). As odorant perfusion on the olfactory epithelium of lampreys from prolarval (Zielinski, Fredricks, Mcdonald, & Zaidi, 2005) to spawning (Li, Sørensen, & Gallaher, 1995) stages activates sensory neurons, olfaction is thought to induce motor behavior throughout life. During the transformation from larva to young adult, the peripheral olfactory apparatus becomes well developed with a lamellar olfactory epithelium and an anatomically distinct accessory olfactory organ (Ren et al., 2009). Moreover, compared to other vertebrate species, lampreys have a large proportion of their brain dedicated to processing olfactory inputs, the size of the olfactory bulbs (OB) even exceeding that of the cerebral hemispheres (Nieuwenhuys, 1977). Furthermore, secondary olfactory neurons have extensive projections throughout the prosencephalon (Northcutt & Puzdrowski, 1988).

Our research group has previously identified a neural substrate responsible for generating locomotion in response to olfactory inputs (Derjean et al., 2010). Odorant detection is carried out by olfactory sensory neurons that project to the medial part of the OB (medOB; Green et al., 2017). There, a unique population of projection neurons located inside a single glomerulus (medOB glomerulus) conveys the inputs to the posterior tuberculum (PT; Daghfous et al., 2018; Derjean et al., 2010; Green, Basilious, Dubuc, & Zielinski, 2013; Pérez‐Fernández, Stephenson‐Jones, Suryanarayana, Robertson, & Grillner, 2014). The PT then sends descending dopaminergic and glutamatergic inputs to the mesencephalic locomotor region (MLR; Ryczko et al., 2013, 2016, 2017), a structure known to play a crucial role in the control of locomotion in all vertebrate species tested so far. The MLR then activates reticulospinal (RS) cells (Sirota, Viana Di Prisco, & Dubuc, 2000), which provide the main descending inputs to the central pattern generators for locomotion in the spinal cord.

Recent findings in our lab have revealed that olfactory projections from the rest of the OB (main OB: MOB) can also activate the PT, but via projections to the lateral pallium (Daghfous et al., 2018). The well‐characterized, locomotion‐controlling neural circuits that are part of an axis from the PT to the spinal cord can thus be activated by both a medial (medOB‐PT) and a lateral (MOB‐lateral pallium‐PT) olfactomotor pathways to generate locomotor responses to olfactory cues. These two parallel olfactomotor pathways convey information from the olfactory sensory organ in the periphery, which is activated by various naturally occurring food‐related or reproductive olfactory cues such as amino acids, bile acids, and pheromones (Green et al., 2017). Thus, they may be equally involved in food‐seeking and mate‐finding.

Multiple odorants that can trigger locomotion elicit responses in the medOB (Green et al., 2017). Moreover, the medial olfactomotor pathway is functional throughout life, but odor‐driven behaviors differ among larval, parasitic (Kleerekoper & Mogensen, 1963; Silva, Servia, Vieira‐Lanero, & Cobo, 2013), and spawning (Johnson, Yun, Buchinger, & Li, 2012) developmental stages. Hence, the activity in this neural circuit must be adjusted to various conditions, external and internal. Modulatory mechanisms upstream of the PT could efficiently regulate motor responses to odorants. The medOB would thus be a good target for modulation of the medial olfactomotor pathway.

Here, the possibility that dopamine (DA) could modulate olfactory processing taking place in the medOB was examined. Dopaminergic processes and cells were previously observed in the OB of lampreys (Barreiro‐Iglesias, Villar‐Cervino, Anadon, & Rodicio, 2009; Fernandez‐Lopez, Sobrido‐Camean, Anadon, Rodicio, & Barreiro‐Iglesias, 2017; Pierre, Mahouche, Suderevskaya, Reperant, & Ward, 1997; Pierre, Rio, Mahouche, & Repérant, 1994; Pombal, El Manira, & Grillner, 1997), as in every other vertebrate studied (Smeets & Gonzalez, 2000). Also, cells expressing D1 or D2 receptors have been observed in the OB (Pérez‐Fernández, 2013; Pérez‐Fernández et al., 2014). DA transmission may thus modulate odor processing in lampreys, such as in other vertebrates. In mammals for instance, DA modulation in the OB is typically associated with olfactory discrimination learning (Escanilla, Yuhas, Marzan, & Linster, 2009; Pavlis, Feretti, Levy, Gupta, & Linster, 2006; Tillerson et al., 2006; Wei, Linster, & Cleland, 2006), and it has also been shown to regulate reproductive behaviors such as mating, parturition, and suckling (Kendrick, Keverne, Chapman, & Baldwin, 1988; Keverne, Lévy, Guevara‐Guzman, & Kendrick, 1993; Serguera, Triaca, Kelly‐Barrett, Banchaabouchi, & Minichiello, 2008). Hence, DA transmission could be involved in the modulation of olfactory processing inducing odor‐driven behaviors in lampreys. This study examines the presence of DA immunoreactivity in the OB and the modulatory actions of DA in the medOB on olfactomotor transmission.

## 2 MATERIALS AND METHODS

Experiments were performed on 72 larvae, 11 newly transformed adults, and 23 spawning‐phase adult sea lampreys (*Petromyzon marinus*) of both sexes. Larval and newly transformed specimens were collected from the Pike River (Pike River, QC, Canada) and the Morpion Stream (Notre‐Dame‐de‐Stanbridge, QC, Canada). The Vermont *US Fish and Wildlife Service* collected spawning‐phase adults. All procedures conformed to the guidelines of the Canadian Council on Animal Care and were approved by the Université de Montréal and the Université du Québec à Montréal ethics and animal care committees. Care was taken to minimize the number of animals used and their suffering.

### 2.1 Anatomical experiments

Anatomical experiments were performed to analyze the distribution of DA+ and tyrosine hydroxylase (TH+) somata and processes in the OB. Under tricaine methanesulfonate anesthesia (MS‐222, 200 mg/L; Sigma Chemical), the animals were decapitated and their brain was isolated in vitro in cold, oxygenated (100% O_2_) Ringer's solution with the following composition (in mM): NaCl, 130.0; KCl, 2.1; CaCl_2_, 2.6; MgCl_2_, 1.8; HEPES, 4.0; dextrose, 4.0; NaHCO_3_, 1.0, adjusted to a pH of 7.4 with NaOH.

The brain tissue at the site of tracer injection was lesioned beforehand with an entomological needle to precisely cut the axons, allowing them to pick up the tracer. Biocytin crystals (Sigma‐Aldrich, St. Louis, MO) were inserted in the lesioned area to dissolve. The brains were then kept overnight under Ringer's solution perfusion at 8°C to allow transport of the tracer to the cell body. To label projection neurons of the medOB, biocytin was injected in the PT. The roof of the caudal part of the third ventricle was cut open along the midline to gain access to the PT. Biocytin injections were also performed in the medOB to retrogradely label neurons projecting to this area.

### 2.2 Histology

#### 2.2.1 Tyrosine hydroxylase immunofluorescence

The brains were fixed by immersion in 4% paraformaldehyde in phosphate‐buffered saline (PBS; 0.1 M, pH 7.4 with 0.9% NaCl) for 24 hours at 4°C, then rinsed in PBS and incubated in a 20% sucrose solution in PBS overnight for cryoprotection. The tissue was frozen in 2‐methylbutane at −50°C and cut transversally with a cryostat. The sections (25 μm thickness) were collected on ColorFrost Plus microscope slides (Thermo Fisher Scientific) and allowed to dry on a warming plate at 37°C for a minimum of 12 hours.

The sections were rinsed (three 10‐min immersions) in PBS, immerged for 1 hour in a permeabilizing solution (normal goat serum 10%, Triton X‐100 0.3%, in PBS), and incubated overnight at 4°C with a primary antibody targeting TH (rabbit anti‐TH, Millipore, Cat# AB152, RRID:AB_390204) diluted 1:400 in the permeabilizing solution. The next day, the sections were rinsed and incubated 1 hour at room temperature with a goat anti‐rabbit antibody conjugated to Alexa Fluor 594 (Molecular Probes, Cat# A‐11012, RRID: AB_141359) diluted 1:400 in the permeabilizing solution. The sections were then rinsed, mounted with Vectashield® (with or without DAPI), and stored in the dark at 4°C.

#### 2.2.2 Dopamine immunofluorescence

The brains were fixed by immersion in 2% glutaraldehyde (pH 7.4) in Tris‐buffered saline with low sodium metabisulfite (Tris 0.05 M with 0.1% sodium metabisulfite and 0.8% NaCl, pH 7.4) for 60 min at 4°C. The fixed brains were then incubated in a 20% sucrose solution in Tris‐buffered saline with low sodium metabisulfite overnight for cryoprotection, frozen in 2‐methylbutane at −50°C, and cut transversally with a cryostat. The sections (25 μm thickness) were collected on ColorFrost Plus microscope slides (Thermo Fisher Scientific) and dried on a warming plate at 37°C for a minimum of 12 hours.

The sections were first rinsed in Tris‐buffered saline with high sodium metabisulfite (TBS‐m: Tris 0.05 M with 1.0% sodium metabisulfite, pH 7.4) and incubated in a reducing solution (sodium borohydride 0.2% in Tris‐buffered saline 0.05 M with 0.9% NaCl, pH 7.4) for 45 min to decrease autofluorescence induced by the glutaraldehyde fixation. The glass slides were rinsed again and immerged in a permeabilizing solution (normal goat serum 10%, Triton X‐100 0.3%, in TBS‐m) for 60 min before overnight incubation at 4°C with a monoclonal mouse anti‐DA antibody (Millipore, Cat# MAB5300, RRID:AB_94817) diluted 1:300 in the permeabilizing solution. The next day, the sections were rinsed and incubated 1 hour with a goat anti‐mouse antibody conjugated to Alexa Fluor 594 (Jackson ImmunoResearch Labs, Cat# 115–585‐146, RRID:AB_2338881) diluted 1:200 in the permeabilizing solution at room temperature. Sections were then rinsed, mounted with Vectashield® (with or without DAPI) and stored in the dark at 4°C.

#### 2.2.3 Antibody characterization

The antibodies used in this study are listed in Table 1. The rabbit anti‐TH antibody has been used reliably on lamprey tissue in independent studies examining the presence of DA neurons (Barreiro‐Iglesias, Villar‐Cervino, Villar‐Cheda, Anadon, & Rodicio, 2008; Robertson et al., 2012). Additionally, our research group has previously used this antibody in lampreys and salamanders (Ryczko et al., 2013, 2016).

**Table 1:** Antibodies used in this study

	Antibody	Immunogen	Source	Dilution
Primary antibodies	Anti‐dopamine antibody, clone K56A	Dopamine‐glutaraldehyde‐BSA	Chemicon, Cat# MAB5300, RRID: AB_94817, raised in mouse, monoclonal	1:300
	Anti‐tyrosine hydroxylase antibody	Denatured tyrosine hydroxylase from rat pheochromocytoma (denatured by sodium dodecyl sulfate)	Chemicon, Cat# AB_94817, RRID: AB_390204, raised in rabbit, polyclonal	1:400
Secondary antibodies	Goat anti‐rabbit IgG (H + L) cross‐adsorbed secondary antibody, Alexa Fluor 594	Rabbit IgG (H + L)	Invitrogen, Cat# A‐11012, RRID: AB_2534079, raised in goat, polyclonal	1:400
	AffiniPure goat anti‐mouse IgG (H + L), Alexa Fluor 594	Mouse IgG (H + L)	Jackson ImmunoResearch Labs, Cat# 115–585‐146, RRID: AB_2338881, raised in goat, polyclonal	1:200
Other	Isolectin GS‐IB4, Alexa Fluor 488	N/A	Life Technologies, Cat# I21411, RRID: AB_2314665, raised in *Griffonia simplicifolia*	1:100
	Streptavidin, Alexa Fluor 488	N/A	Invitrogen, Cat# S11223, RRID: AB_2336881	1:200
	Streptavidin, Alexa Fluor 350	N/A	Invitrogen, Cat# S11249	1:200

Abbreviations: BSA, bovine serum albumin; IgG, immunoglobulin G.

The pattern of labeling of the mouse antibody targeting DA in our material corresponded closely to that reported with other DA antibodies in the lamprey (Abalo, Villar‐Cheda, Anadon, & Rodicio, 2005; Barreiro‐Iglesias, Villar‐Cervino, Anadon, & Rodicio, 2008; Pierre et al., 1997). Cross‐reactivity of the mouse anti‐DA antibody was determined by the manufacturer (Millipore) using an ELISA test with the following compounds: DA‐glutaraldehyde (G)‐bovine serum albumin (BSA) 1; Tyrosine‐G‐BSA 1:36,000; L‐DOPA‐G‐BSA 1:10,000; Noradrenaline‐G‐BSA 1:>50,000; Adrenaline‐G‐BSA 1:>50,000. The specificity of the fluorescent secondary antibodies was verified by omitting the primary antibody from the procedures. In every case, no labeling was obtained under these conditions.

#### 2.2.4 Additional labeling

Streptavidin conjugated to Alexa Fluor 488 or 350 (diluted 1:200, S11223 or S11249, Thermo Fisher Scientific) was added to the secondary antibody solution to visualize biocytin. The primary olfactory afferents were stained with *Griffonia simplicifolia* isolectin B4 (GSIB4), which binds to galactosyl residues present on axons of olfactory sensory neurons, as previously done by others (Tobet, Chickering, & Sower, 1996) and us (Daghfous et al., 2018). This labeling was carried out after the immunofluorescence protocol by incubating the slides with GSIB4 conjugated to Alexa Fluor 488 (I21411, Life Technologies) diluted 1:100 in the appropriate rinsing solution (TBS‐m or PBS) for 60 min at room temperature. The sections were then rinsed and fixed in 4% paraformaldehyde in PBS for 1 hour at room temperature before they were rinsed again and mounted.

#### 2.2.5 Fluorescence microscopy

The sections were observed and photographed on an E600 epifluorescence microscope equipped with a DMX1200 digital camera driven by the Automatic Camera Tamer software (Nikon Canada, Mississauga, ON, Canada). Photoshop CS5 software (Adobe Systems, San Jose, CA) was used to merge the photomicrographs. Photomicrographs taken with a 20X objective were assembled with the *Photomerge* function in Photoshop CS5. The sections were then drawn from the photomontage. The outline of the sections and OB glomeruli (GSIB4 labeling), as well as DA+ processes and cells were then drawn with precision on Illustrator CS5 software (Adobe Systems). The accuracy of the illustrations was validated under the microscope. The red labeling was transformed to magenta in Photoshop CS5. The only other changes made to the images were brightness and contrast adjustments.

### 2.3 Electrophysiological experiments

Whole brain preparations from larval specimens were isolated in vitro as described above and pinned down in an experimental chamber (total volume = 50 mL) continuously perfused with a Ringer's solution at a rate of 4 mL/min and maintained between 8 and 13°C. A minimum of 60 min of postoperative recovery preceded the electrophysiological recordings. Intracellular recordings of RS cells were performed with sharp glass microelectrodes (filled with KAc 4 M; 80–120 MΩ). The electrical signal was amplified using an Axoclamp 2A amplifier (Axon Instruments, Union City, CA) coupled to a Digidata 1200 (Axon Instruments) and stored on a computer using *Axoscope* software (Axon Instruments, Version 9.2.1.8). The RS cells analyzed in this study had a membrane potential under −70 mV that was stable for a minimum of 15 min after impaling the cell. The larger Müller cells (B1, B3, and B4) were usually the cells that were recorded for reproducibility of the results. The ON was electrically stimulated with homemade glass‐coated tungsten microelectrodes (4–5 MΩ; 30–50 μm tip exposure) connected to a Grass S88 stimulator coupled to a Grass PSIU6 photoelectric isolation unit for controlling the stimulation intensity (Astro‐Med Inc., Longueuil, Canada). Trains of 1–3 pulses (50 Hz, 2 ms duration, 2–30 μA) were applied during resting activity of RS cells. To prevent desensitization, a minimum of 50 s was allocated between each train and the stimulation intensity was kept at threshold level to generate responses.

#### 2.3.1 Drug application

Drugs were pressure ejected through glass micropipettes (tip diameter: 10–20 μm) positioned directly in the OB tissue. The ejection micropipette was inserted from the lateral OB and pressure ejections were delivered by a *Picospritzer II* (Parker Hannifin, General Valve Division, Fairfield, NJ). An average of 31 ± 30 pulses of 20–30 ms duration at about 4 psi were delivered, yielding mean ejection volumes of 1.96 ± 1.90 nL. Adding the inactive dye Fast Green to the drug solution allowed to visually monitor the diffusion in the tissue. The injections were centered on the medOB and did not exceed 300 μm in diameter.

The following drugs were used in this study: Dopamine hydrochloride (1.0 mM; nonselective DA receptor agonist; Sigma‐Aldrich, St. Louis, MO); Dihydrexidine hydrochloride (0.1 mM; selective D1 receptor agonist; Tocris Bioscience, Bristol, UK); (−)‐Quinpirole hydrochloride (0.1 mM; selective D2 receptor agonist; Sigma‐Aldrich, St. Louis, MO); R(+)‐SCH‐23390‐hydrochloride (0.5 mM; selective D1 receptor antagonist; Sigma‐Aldrich, St. Louis, MO); Raclopride (0.1 mM; selective D2/D3 receptor antagonist; Tocris Bioscience, Bristol, UK); Gabazine (0.1 mM; selective GABA_A_ receptor antagonist; Tocris Bioscience, Bristol, UK). All drugs were dissolved in Ringer's solution and kept at −20°C (or 4°C for less than 7 days) until application.

#### 2.3.2 Data analysis

Electrophysiological data were analyzed with Spike2 software (Cambridge Electronic Design, Version 5.19) and a homemade script for excitatory postsynaptic potentials (EPSPs). Statistical analyses were carried out on Sigmaplot (Systat Software Inc., Version 11.0). One‐way ANOVA for repeated measures or Friedman ANOVA on ranks for repeated measures were used and followed by multiple comparison procedures (Holm‐Sidak or Tukey) to test equality of means in the different treatments (Control – Drug – Washout). For all statistical analyses, a significance level of .05 was adopted. Results are presented as the mean ± *SD*.

## 3 RESULTS

### 3.1 Dopamine immunofluorescence in the olfactory bulb

**Figure 1:**
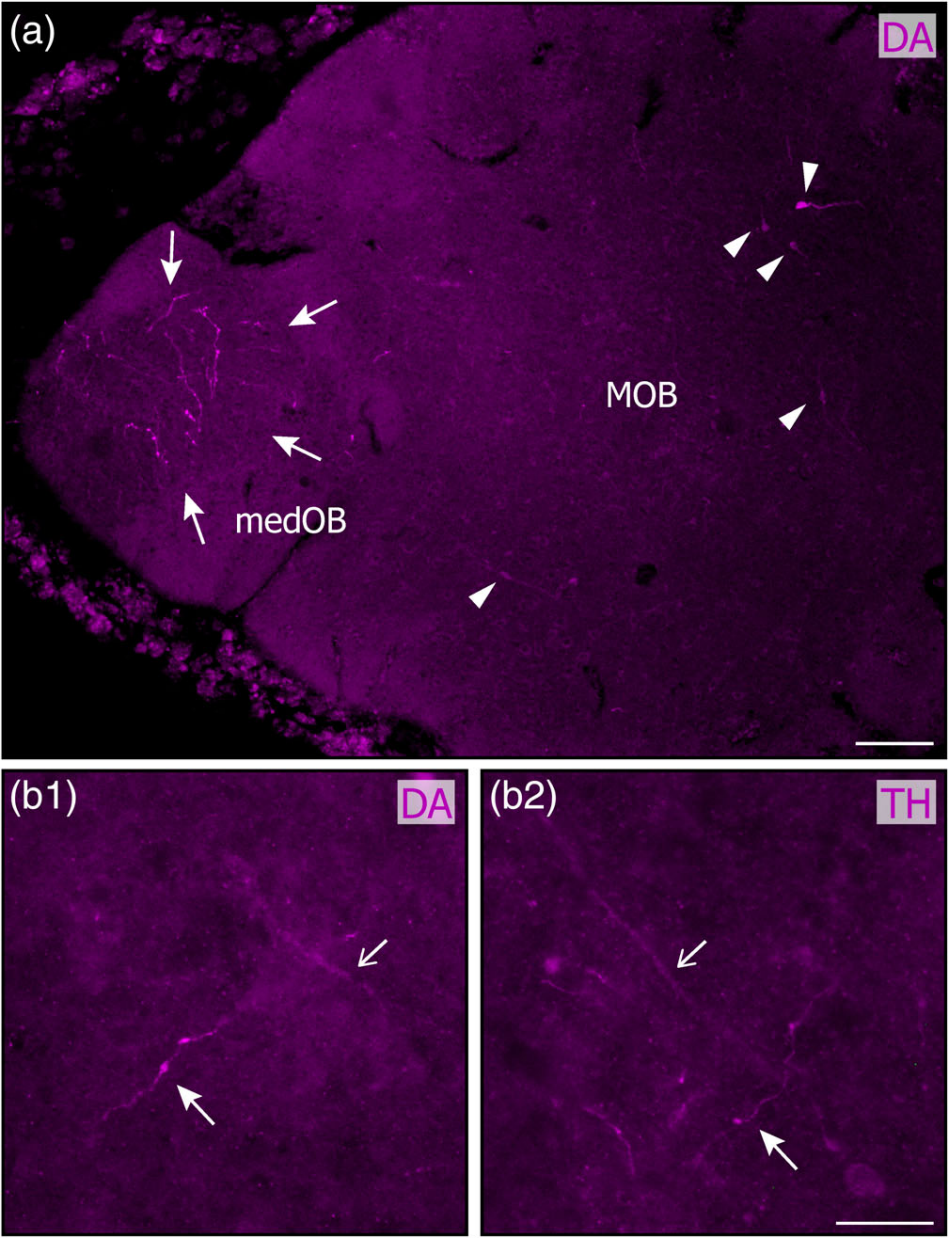
Dopaminergic processes in the olfactory bulb of lampreys. (a) Low magnification photomicrograph showing immunofluorescence against dopamine (DA) on an olfactory bulb transverse section from a spawning‐phase adult sea lamprey. Strongly labeled DA+ processes are mostly present in the medial part of the olfactory bulb (medOB) particularly in the area delimited by arrows. Immunoreactive somata (arrowheads) with weakly labeled processes are observed in the main olfactory bulb (MOB). (b) Photomicrographs illustrating two types of processes in the same picture frame (weakly labeled: empty arrow, strongly labeled: solid arrow) in the granular layer, after DA (b1) and tyrosine hydroxylase (TH; b2) immunofluorescence. Scale bar for (a): 100 μm; scale bar for (b): 25 μm [Color figure can be viewed at http://wileyonlinelibrary.com]

An immunofluorescence procedure was performed in 12 larval, six newly transformed adult, and 11 spawning‐phase adult lampreys of both sexes to analyze the distribution of DA and TH immunoreactivity in the OB. The most notable feature brought to light was the presence of two distinct types of processes that were differentially distributed in the OB (Figure 1).

**Figure 2:**
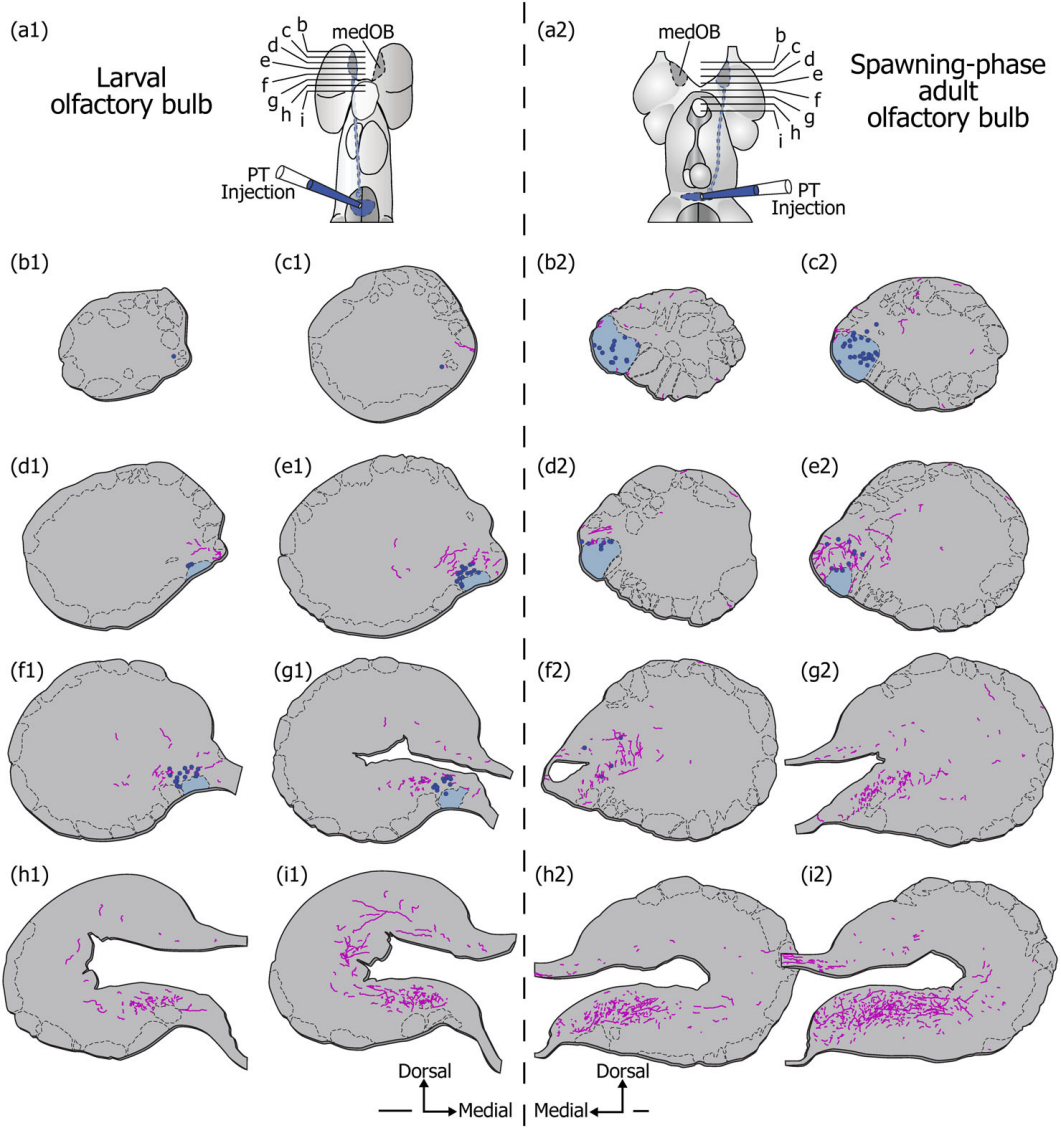
Localization of strongly labeled dopaminergic processes in the olfactory bulb of larval and adult lampreys. Drawings of serial transverse representative hemisections of the larval (left) and spawning‐phase adult (right) olfactory bulb at corresponding rostrocaudal levels. The schematic dorsal view of the prosencephalon (a) shows the rostrocaudal level of the sections represented (b–i). Strongly labeled dopaminergic (DA) processes were drawn as magenta lines and the olfactory glomeruli, visualized with GSIB4 labeling, are delimited with black dashed lines. Biocytin injection in the posterior tuberculum (PT) backfilled projection neurons (enlarged blue dots) in the medial olfactory bulb (medOB). The medOB glomerulus (colored in blue) corresponds to the overlapping of the GSIB4 labeling and the arborization of the dendrites from the backfilled projection neurons. Scale bars: 100 μm; distance between sections: 25 μm (larva) and 75 μm (spawning‐phase adult) [Color figure can be viewed at http://wileyonlinelibrary.com]

**Figure 3:**
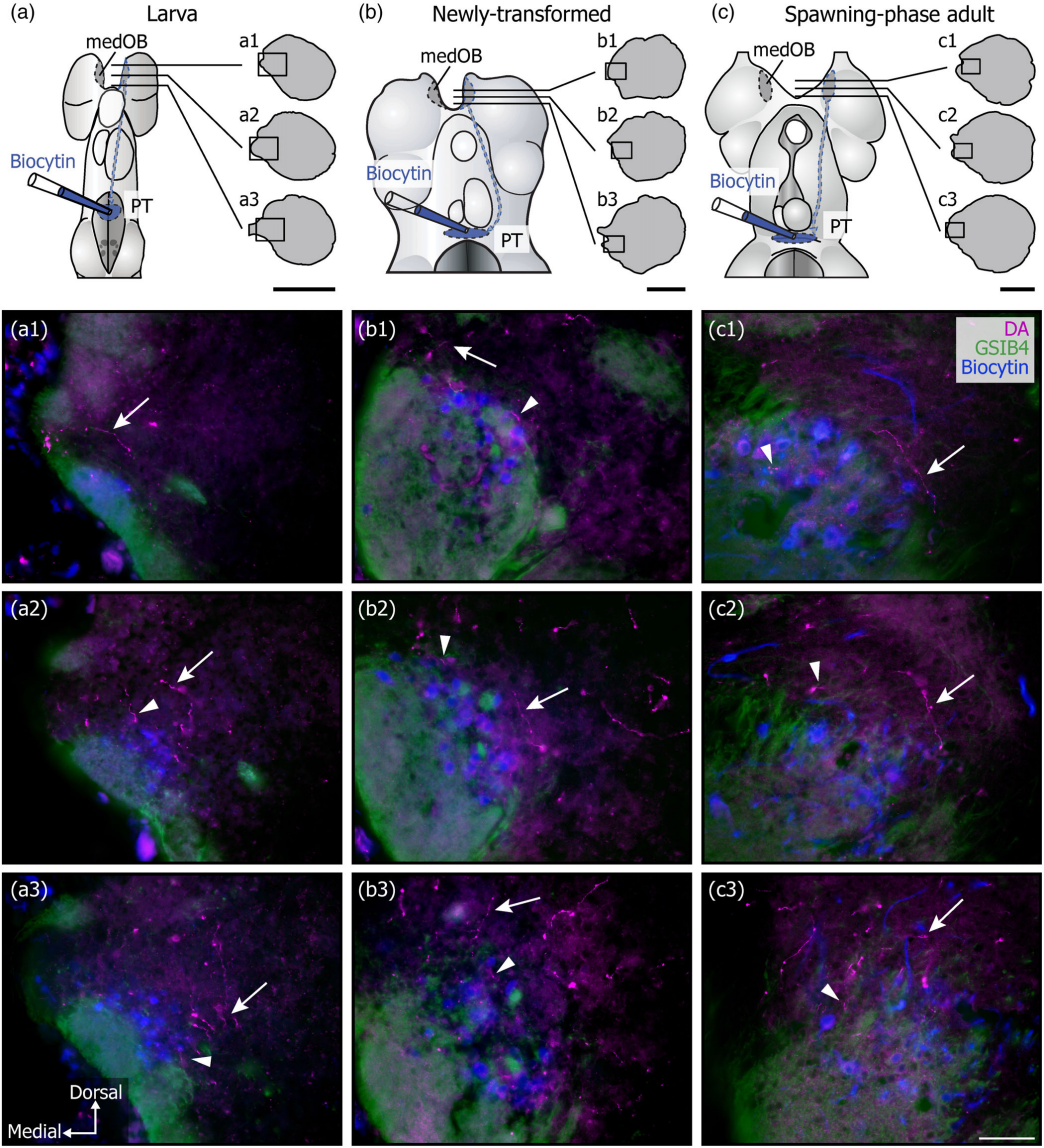
Strongly labeled dopaminergic processes in the medOB. Photomicrographs of the medial olfactory bulb at three different developmental stages: larval (a), newly transformed (b) and spawning‐phase adult (c), at three different rostrocaudal levels (1, 2, 3). The top three panels contain a schematic dorsal view of the prosencephalon showing the levels at which the photomicrographs were taken (black boxes on the corresponding hemisection drawings). Photomicrographs are the result of merging three different labelings: dopamine (DA) immunofluorescence (magenta); GSIB4 binding to primary olfactory afferents that form the glomeruli (green); and biocytin labeling (blue) of medOB projection neurons and their dendrites following injection in the posterior tuberculum (PT). Strongly labeled DA+ processes are seen within (arrowheads) and in the periphery of (arrows) the medOB glomerulus, close to projection neurons and primary olfactory afferents. Scale bars for hemisection drawings (a–c): 500 μm; scale bar for photomicrographs (a1–c3): 50 μm [Color figure can be viewed at http://wileyonlinelibrary.com]

**Figure 4:**
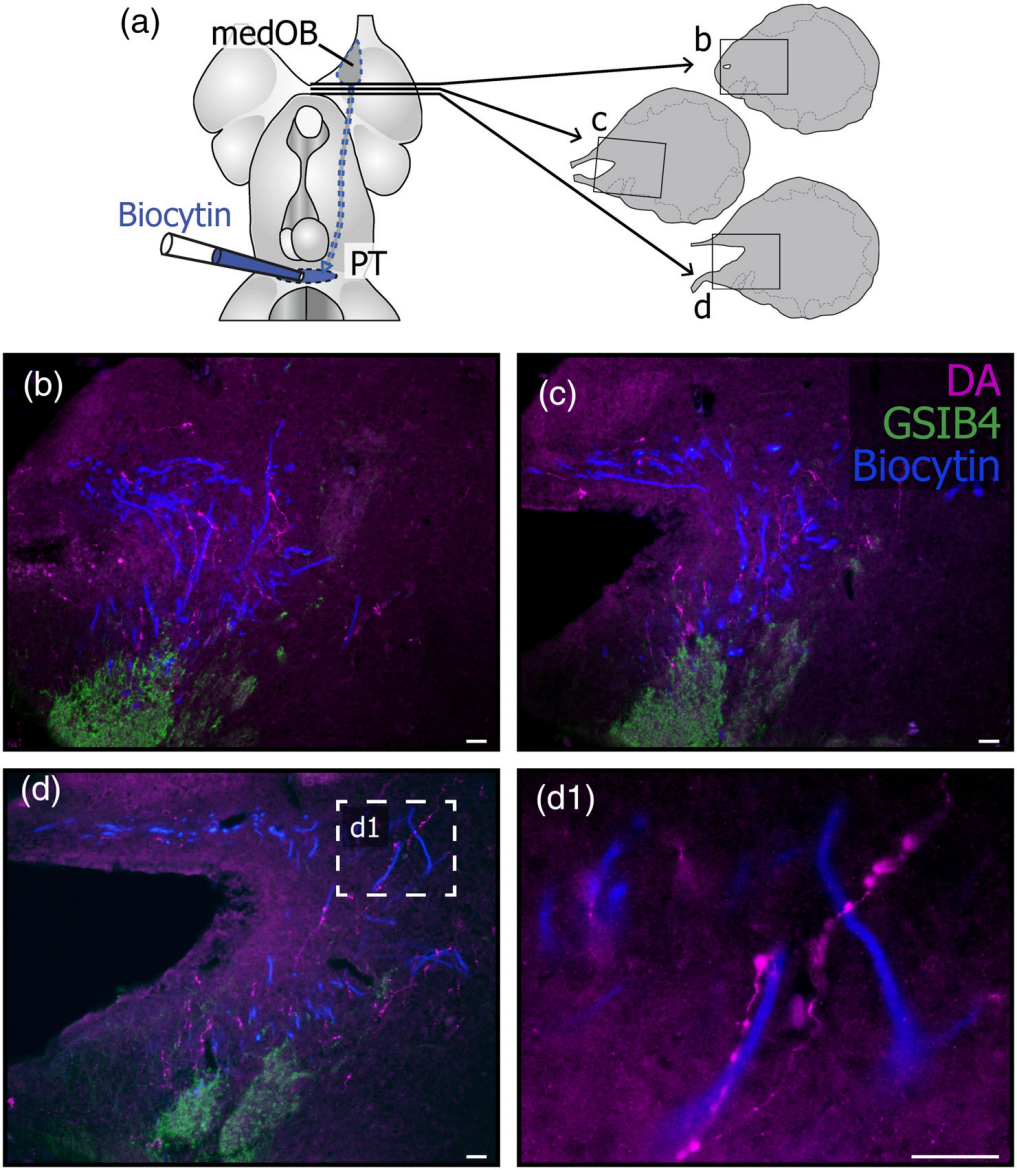
Descending axons from medOB projection neurons and their relationship with strongly labeled dopaminergic (DA) processes. Projection neurons of the medOB send large diameter descending axons (blue in b–d) to the posterior tuberculum (PT). Those descending axons travel alongside strongly labeled DA+ processes (magenta in b–d) presumably ascending to the OB. Primary olfactory afferents were labeled in green with GSIB4. (a) Schematic dorsal view of the spawning‐phase adult prosencephalon showing the biocytin injection site and the rostrocaudal levels (black lines) corresponding to transverse hemisections illustrated in (b–d). (b, c) Photomicrographs showing descending large axons (blue) that originate from more rostrally located projection neurons of the medOB. Upon leaving the medOB caudally, these axons are accompanied by DA+ processes. (d) Photomicrograph of a transverse section, slightly more caudal than those in (b, c). The large axons from the medOB projection neurons are now divided into a dorsal and a ventral tract, both containing varicose, strongly labeled DA+ processes. (d1) High magnification of the boxed area in (d) shows the proximity of the two types of processes. Scale bars: 25 μm [Color figure can be viewed at http://wileyonlinelibrary.com]

The processes from a first type were varicose and strongly labeled. They were readily seen under epifluorescence microscopy, even at low magnification (Figure 1). These strongly labeled processes were preferentially located medially and caudally within the OB, although scarce and isolated processes were found in all bulbar regions (Figures 1 and 2). Interestingly, these processes were found at all life stages, from larvae to spawning‐phase adults, with very similar characteristics and distribution in each case. Moreover, processes with the same characteristics were labeled after immunofluorescence against TH (Figure 1b). Most of the strongly labeled DA+ and TH+ processes were located around and inside the medOB, close to projection neurons and primary olfactory afferents. Projection neurons of the medOB were retrogradely labeled to examine their relationship with DA+ processes (Figures 2, 3, 4). Following injection in the PT, biocytin‐labeled projection neurons were restricted to the medOB, although in larvae most were clustered more caudally than in adults (Figures 2 and 3). Varicose DA+ processes were observed in the periphery and inside of the medOB, among the GSIB4‐labeled primary olfactory afferents and in close proximity to projection neuron somata and dendrites. Caudal to the medOB, the DA+ processes coursed along the axons of the medOB projection neurons (Figure 4). It was impossible to follow them distinctly in the septum, which contains a dense plexus of similarly labeled DA+ processes. No DA+ processes colabeled by biocytin injection in the PT were seen in the OB. The combined staining of primary olfactory afferents, projection neurons, and DA in the medOB suggests strongly that DA+ processes innervate elements of the medOB at every developmental stage.

**Figure 5:**
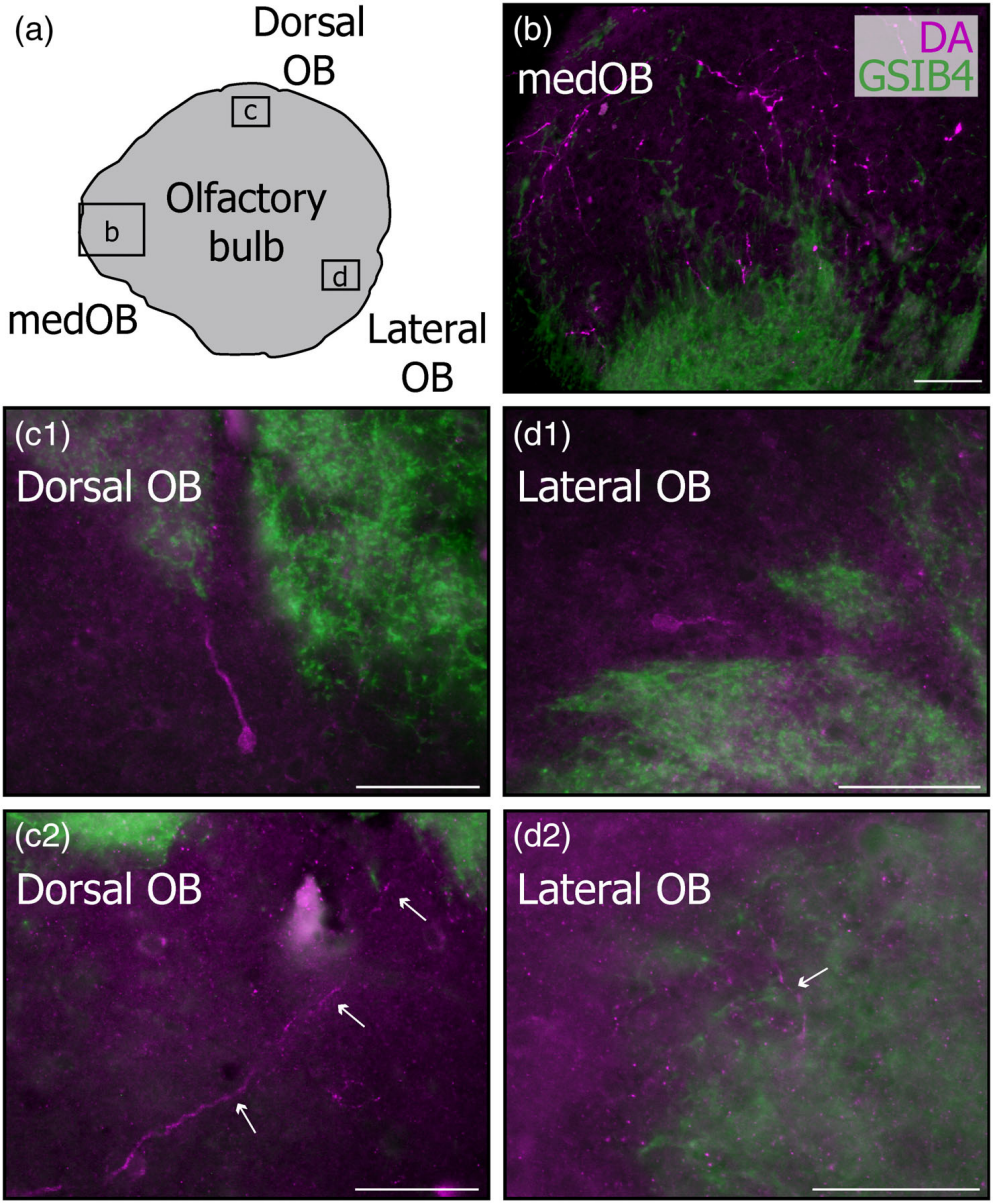
Dopaminergic processes in glomerular territories of the olfactory bulb. (a) Drawing of a transverse section of a spawning‐phase adult right olfactory bulb, illustrating the approximate localizations (boxed areas) of the photomicrographs in (b–d), which were taken from different animals. Dopaminergic (DA) cells (c1, d1) and processes (b, c2, d2; arrows) are seen close to olfactory glomeruli. (b–d) Merged photomicrographs showing labeled olfactory primary afferents in glomeruli (GSIB4, green) in combination with DA immunofluorescence (magenta). Scale bars: 50 μm [Color figure can be viewed at http://wileyonlinelibrary.com]

In the OB of newly transformed and spawning‐phase adults, DA immunofluorescence yielded additional labeling. Notably in adult lampreys, a second type of DA+ processes were detected (Figures 1 and 5). These processes were in sharp contrast with those previously described: they were weakly labeled, did not display varicosities, and were spread homogeneously across the granular layer. Isolated processes also occasionally reached the glomerular layer (Figure 5c2,d2).

**Figure 6:**
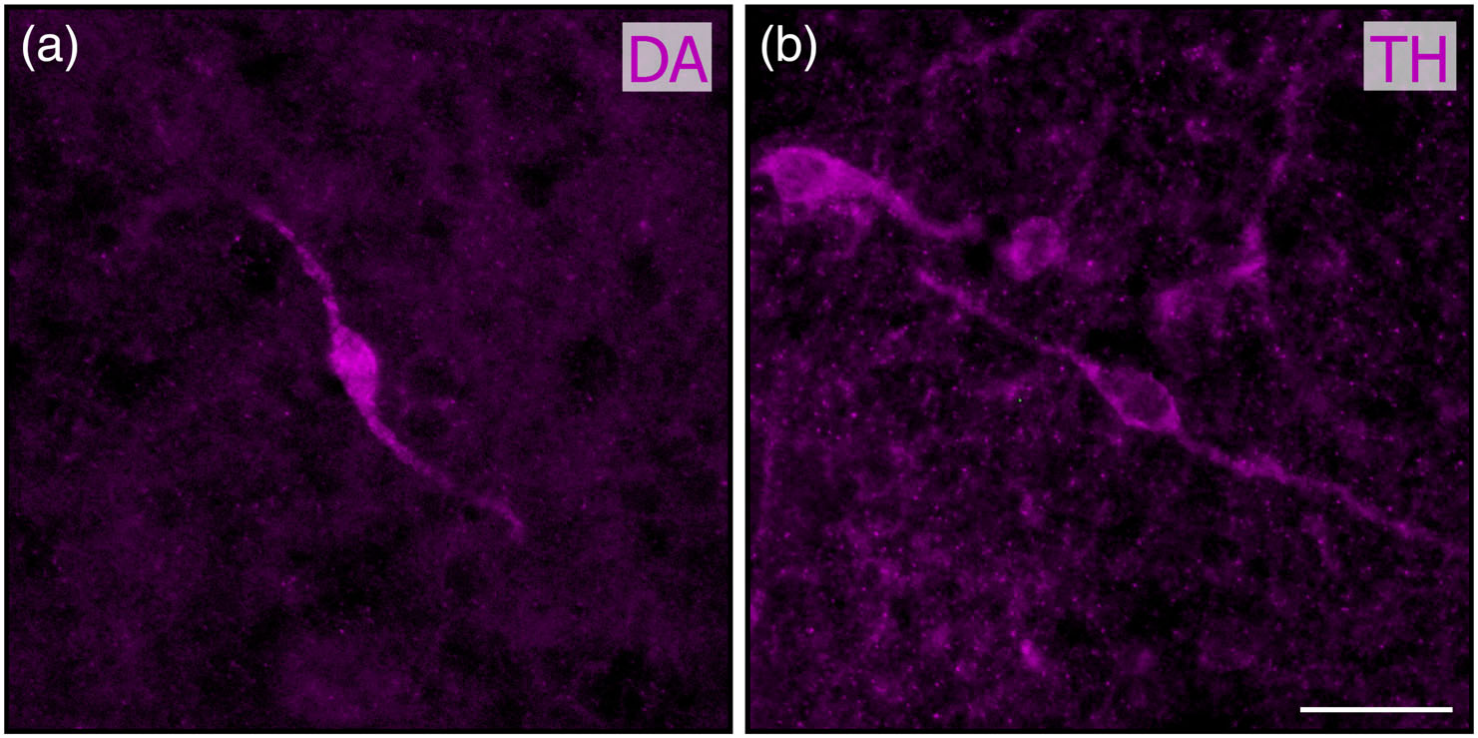
Dopamine and tyrosine hydroxylase immunoreactive cell bodies in the olfactory bulb. High magnification photomicrographs showing neuronal cell bodies labeled after immunofluorescence against dopamine (DA; a) and tyrosine hydroxylase (TH; b) in the granular layer of the olfactory bulb in spawning‐phase adults. Scale bar: 25 μm [Color figure can be viewed at http://wileyonlinelibrary.com]

In addition, processes with same characteristics were often seen originating from local cell bodies, which were also exclusively observed in newly transformed and spawning phase adults. These somata had a similar, weak labeling intensity, and were small‐sized (10–15 μm), round or ovoid and often bipolar (Figure 6). From the most caudal to the most rostral levels, they were homogeneously scattered throughout the granular cell layer. They were occasionally seen bordering glomeruli in the dorsal and lateral OB, extending a process into the glomerular neuropil (Figure 5c1,d1). After immunofluorescence against TH, very similar neurons were consistently observed; they showed a more intense labeling, making them easier to visualize. They exhibited similar size, shape, and distribution when compared to the DA+ cell bodies described above, the only difference being that a greater number of TH somata could be visualized. Moreover, despite the absence of DA+ somata and weakly labeled processes in larval specimens, both were observed consistently in larvae following TH immunofluorescence.

### 3.2 Dopaminergic afferents to the medial olfactory bulb

**Figure 7:**
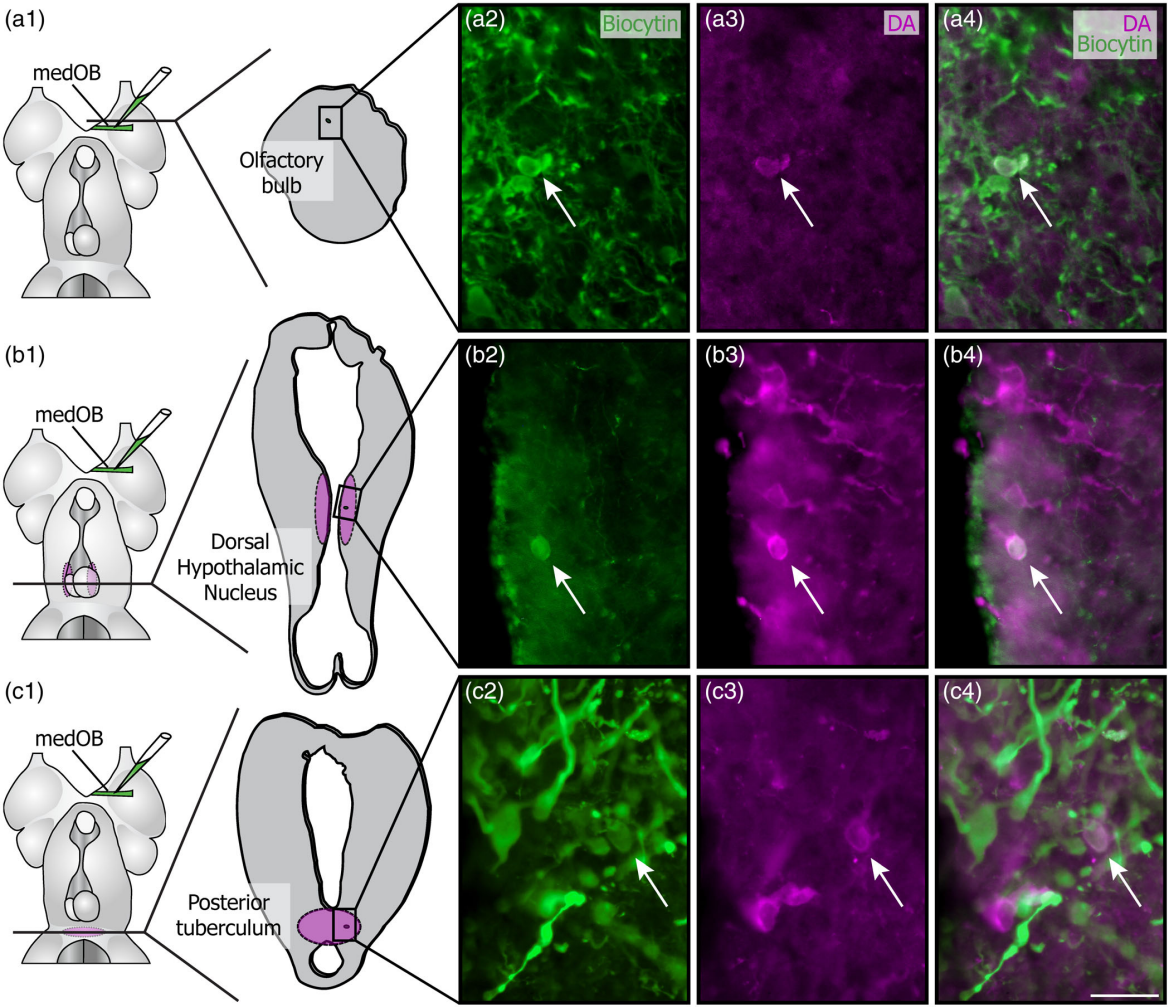
Origins of the dopaminergic projections to the medOB in spawning‐phase adults. Retrograde axonal tracing from the medial olfactory bulb (medOB) was combined with dopamine (DA) immunofluorescence to label neurons responsible for the DA+ processes in the medOB. The schematic dorsal views of the spawning‐phase adult lamprey prosencephalon (a1–c1) illustrate the biocytin injection site and the levels at which double‐labeled neurons were observed. The drawings of the corresponding transverse sections also show the frame of the photomicrographs to the right. Pictures in a4, b4, and c4 are a merge of a2‐a3, b2‐b3, and c2‐c3, respectively. These images show examples of neurons (arrows) in the olfactory bulb (a), dorsal hypothalamic nucleus (b), and posterior tuberculum (c) labeled by both DA immunofluorescence and biocytin injection in the medOB. Scale bars: 25 μm [Color figure can be viewed at http://wileyonlinelibrary.com]

**Figure 8:**
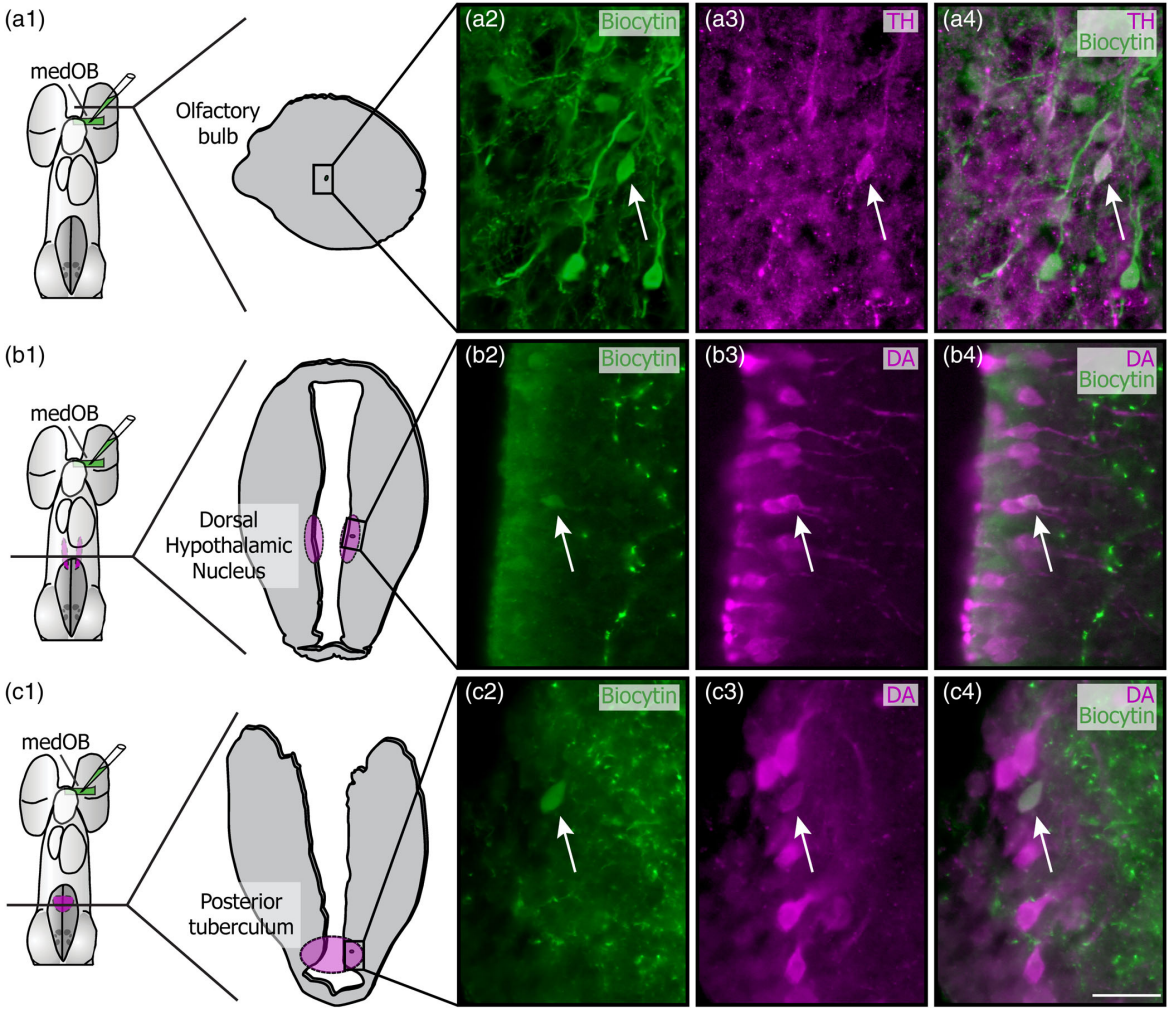
Origins of the dopaminergic projections to the medOB in larvae. Retrograde axonal tracing from the medial olfactory bulb (medOB) was combined with tyrosine hydroxylase (TH; a) or dopamine (DA; b, c) immunofluorescence to label neurons responsible for the DA+ processes in the medOB. Immunofluorescence against TH was used in the olfactory bulb of larvae because immunofluorescence against DA did not yield any labeling of local cell bodies. The schematic dorsal views of the larval prosencephalon (a1–c1) illustrate the biocytin injection site and the levels at which double‐labeled neurons were observed. The drawings of the corresponding transverse sections also show the frame of the photomicrographs to the right. Pictures in a4, b4, and c4 are a merge of a2‐a3, b2‐b3, and c2‐c3, respectively. These images show examples of neurons (arrows) in the olfactory bulb (a), dorsal hypothalamic nucleus (b), and posterior tuberculum (c) labeled by both DA (or TH) immunofluorescence and biocytin injection in the medOB. Scale bars: 25 μm [Color figure can be viewed at http://wileyonlinelibrary.com]

In the adult OB, the weakly labeled DA+ processes could stem from local neurons, as they displayed the same labeling intensity than the DA+ cell bodies and their associated processes in the granular layer. However, no labeled cell bodies were found to be as intensely labeled as the strongly labeled processes. It is possible that an extrinsic source of DA innervation to the OB exists. To determine the presence of such an extrinsic source of DA+ processes, biocytin injections in the medOB were combined with immunofluorescence targeting DA. In spawning‐phase adults (*n* = 12), retrogradely labeled DA+ neurons were detected in the granular layer of the OB (Figure 7a), in the dorsal hypothalamic nucleus (DHN, Figure 7b), and in the PT (Figure 7c). In larvae (*n* = 13), DA+ neurons were also retrogradely labeled in the DHN (Figure 8b) and the PT (Figure 8c) but not in the OB, as they are undetected at this developmental stage (see above). In combination with TH immunofluorescence (*n* = 12 larvae), medOB injections labeled numerous TH+ neurons in the OB (Figure 8a). In the DHN and PT, TH+ neurons were also labeled by biocytin injections and were similar in morphology and localization to those observed with immunofluorescence against DA. The double‐labeled neurons in the DHN were cerebrospinal fluid‐contacting cells. Those observed in the PT were within the DA cell population described as homologous to the mammalian substantia nigra, pars compacta, and ventral tegmental area (SNc/VTA; Pombal et al., 1997).

### 3.3 The effect of dopamine on reticulospinal cell responses induced by olfactory inputs

Our anatomical results altogether suggest that DA+ processes from extrinsic sources (DHN and PT) innervate the medOB in addition to intrinsic sources (OB). The next step was to investigate whether the DA innervation modulates the transmission in the OB. In the larval OB, which did not contain DA+ somata or weakly labeled processes, strongly labeled DA+ processes were more densely localized close to and inside the medOB. Hence, electrophysiological experiments were carried out to characterize the physiological effects of DA on the medial olfactomotor pathway (Derjean et al., 2010). Projection neurons in the medOB send direct output to the PT and to the MLR, which relay the signal to RS cells to generate locomotor activity (Daghfous et al., 2018; Derjean et al., 2010). The recording of RS cells, which act as command cells for locomotion, can monitor this activity. Experiments were performed in the in vitro isolated larval brain to test the effects of DA microinjection in the medOB on RS cell synaptic responses to olfactory nerve (ON) stimulation.

**Figure 9:**
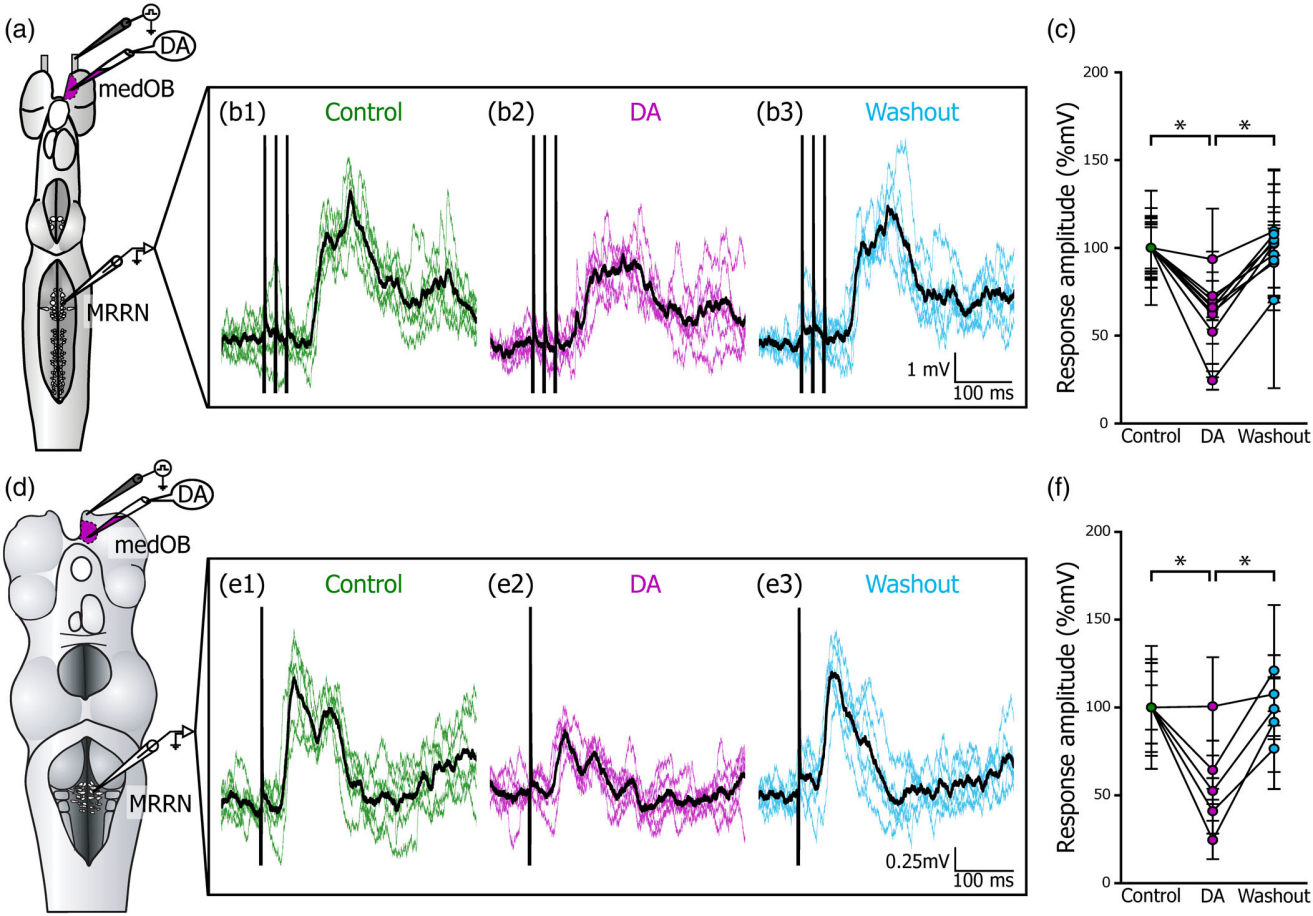
Effect of dopamine injection in the medOB on RS cell responses. The schematic dorsal view of the isolated larval (a) and newly transformed adult (d) brains illustrate the preparation where synaptic responses to electrical stimulation of the olfactory nerve were intracellularly recorded in ipsilateral reticulospinal neurons. (b1–b3, e1–e3) Responses evoked by electrical stimulation (black vertical bars: stimulation artifact) are represented as six superimposed traces (colored) and their mean (thick black trace). Compared to control conditions (b1, e1), responses were decreased following dopamine (DA) injection (1 mM) in the medial olfactory bulb (medOB) (b2, e2), and this effect was reversed after washout (b3, e3). (c, f) Mean response amplitudes during each condition are plotted as a line graph for every recorded RS cell (c, *n* = 9 cells in nine larvae; f, *n* = 5 cells in five newly transformed adults). MRRN, middle rhombencephalic reticular nucleus. *p < .001 [Color figure can be viewed at http://wileyonlinelibrary.com]

Reticulospinal cell responses to electrical stimulation (1–3 pulses at 50 Hz, 2 ms duration, 10–30 μA) of the ON were recorded intracellularly. Local pressure microinjection of DA (1 mM) in the medOB (Figure 9a–c) induced a significant reduction in the amplitude (to 63.4 ± 24.9%, *F* = 43.996, *df* = 2, *p* < .001, *n* = 9 cells in nine larvae) and the area (to 52.8 ± 66.6%, *χ* ^2^ = 42.481, *df* = 2, *p* < .001, *n* = 9 cells in nine larvae) of the EPSPs. The responses recovered to control level after washout (amplitude: *t* = 0. 581, *p* = .563; area: *q* = 0.680, *p* ≥ .05). This experiment was then performed in newly transformed animals to determine if DA modulation also occurs in adults (Figure 9d–f). As in larval animals, the RS cell responses were significantly reduced in amplitude (to 56.6 ± 30.4%; *F* = 29.825; *df* = 2; *p* < .001, *n* = 5 cells in five newly transformed adults) and area (to 43.5 ± 45.4%; *F* = 22.153; *df* = 2; *p* < .001, *n* = 5 cells in five newly transformed adults) by DA microinjection in the medOB. There were no significant differences between control and washout (amplitude: *t* = 0.125, *p* = .901; area: *t* = 0.557, *p* = .580).

**Figure 10:**
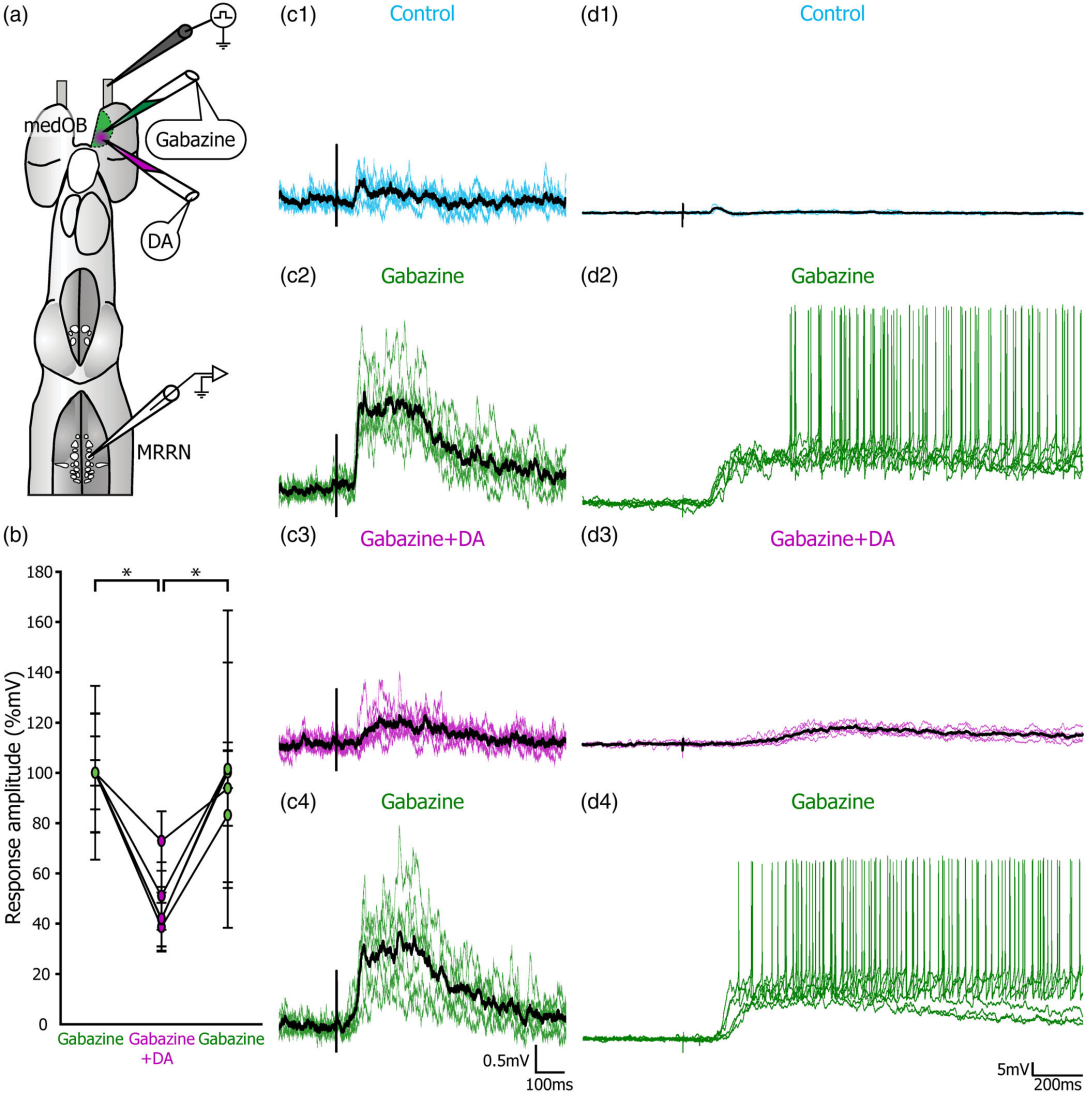
Effect of dopamine injection in the medOB on RS cell responses with prior removal of local medOB GABAergic inhibition with gabazine. (a) Schematic illustration showing the experimental procedure where GABA_A_ receptor antagonist gabazine (0.1 mM) was periodically injected in the medial OB (medOB) to disinhibit olfactory nerve (ON) stimulation‐evoked synaptic responses in reticulospinal (RS) cells. When dopamine (DA) and gabazine are simultaneously injected in the medOB, the mean response amplitude is significantly decreased (*n* = 5 cells in five larvae). (b) Mean response amplitudes during each condition are plotted as a line graph for every recorded RS cell. (c) Illustrates a representative example of RS cell subthreshold responses under control conditions (c1), gabazine injection (c2), gabazine and DA injection (c3), and after washout of DA but still under gabazine (c4). Another example shows RS cell suprathreshold responses under the same conditions (d1–d4). Colored traces are a superimposition of six responses to ON stimulation and their mean is represented by a thicker black trace. Although local injection of gabazine in the medOB amplifies RS cell synaptic responses, a combined DA injection induces a marked decrease of these subthreshold (c) or suprathreshold (d) responses, which is reversed after washout of DA. MRRN, middle rhombencephalic reticular nucleus. *p < .001 [Color figure can be viewed at http://wileyonlinelibrary.com]

We also tested the effects of DA on suprathreshold responses to get a better indication on whether DA also affects motor behavior. Previous work from our group has shown that blocking GABA_A_ receptors in the OB with gabazine amplifies considerably RS cell responses to electrical stimulation of the ON (Daghfous et al., 2018). Therefore, the effects of DA were tested on amplified RS responses to stimulation of the ON (Figure 10). Microinjection of gabazine (0.1 mM) in the medOB markedly amplified RS cell responses to ON stimulation, as previously described (Daghfous et al., 2018). A subsequent DA microinjection in the medOB significantly reduced the synaptic response amplitude (to 49.2 ± 16.8%; *χ* ^2^ = 42.467, *df* = 2, *p* < .001, *n* = 5 cells in five larvae) and area (to 30.2 ± 18.3%; *χ* ^2^ = 46.067, *df* = 2, *p* < .001, *n* = 5 cells in five larvae) (Figure 10b,c) and the responses recovered to control values after DA washout (amplitude: *q* = 0.913, *p* ≥ .05; area: *q* = 1.461, *p* ≥ .05). Moreover, after gabazine microinjection, RS cells often displayed spiking activity in response to ON stimulation (Figure 10d). Under these conditions, microinjection of DA in the medOB could reduce the excitatory responses in RS cells to the point of eliminating spiking activity. Therefore, the local effects of DA in the medOB may significantly affect motor output.

### 3.4 The role of D1 and D2 receptors in modulating olfactomotor transmission

**Figure 11:**
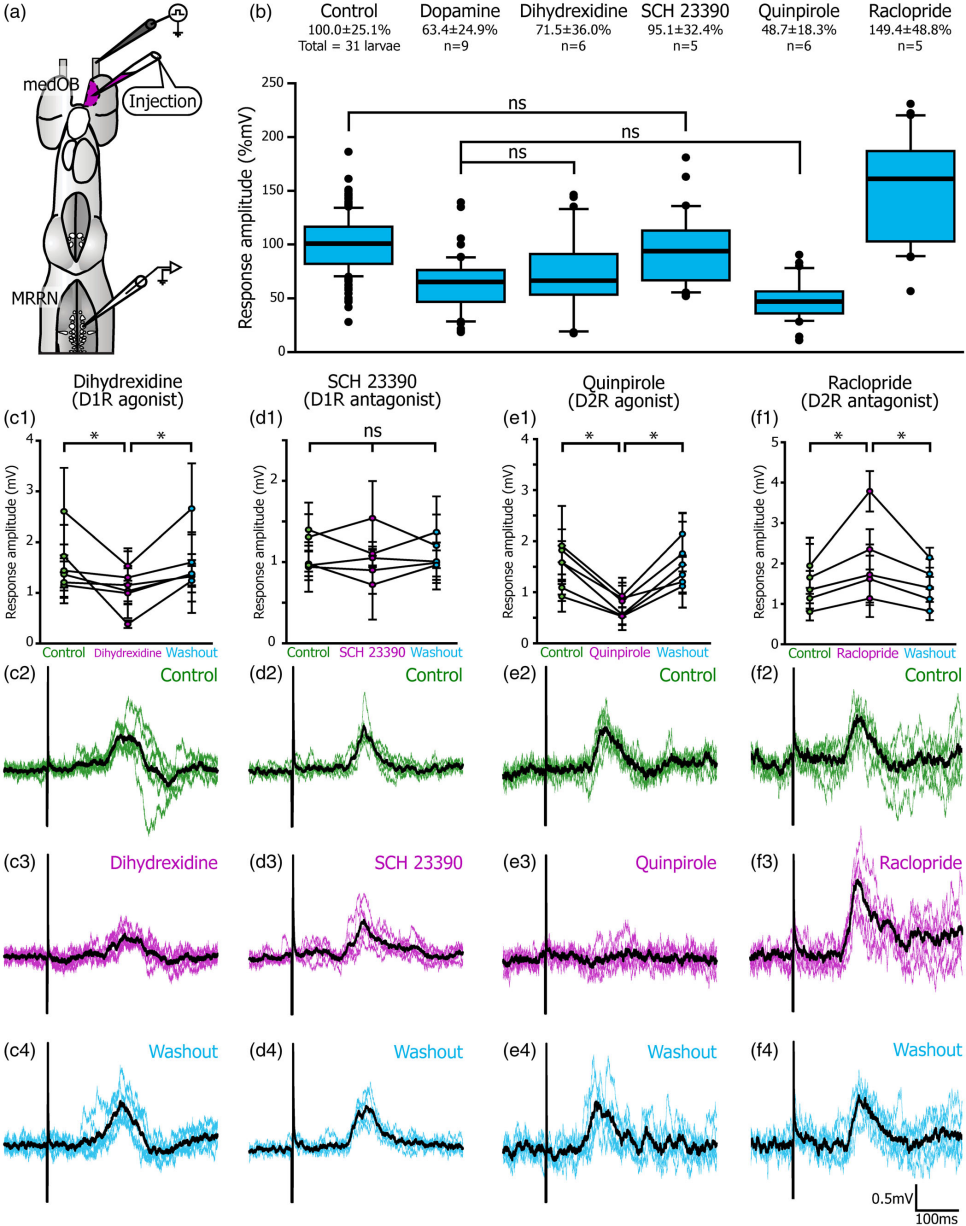
Effects of D1 and D2 receptor agonists and antagonists injection in the medOB on RS cell responses. The physiological effects of selective dopamine (DA) receptor ligands injection in the medial olfactory bulb (medOB) on reticulospinal (RS) cells synaptic responses to olfactory nerve stimulation was studied in isolated larval brains (a). The data were compiled in a boxplot (b) representing the relative response amplitude following local drug injections in comparison with control responses from every recording. In addition, the mean response amplitude of individual RS cells before, during and after injection of selective DA receptor agonists or antagonists was plotted as different line graphs (c1, d1, e1, and f1). Evoked responses from representative recordings are exhibited as six superimposed traces and their mean (thick black trace) during each treatment (c2–c4; d2–d4; e2–e4; f2–f4). (c) D1 receptor agonist dihydrexidine (0.1 mM) decreased the mean response amplitude to 71.5 ± 36.0% of control (*n* = 6 cells in six larvae). (d) D1 receptor antagonist SCH 23390 (0.5 mM) did not produce robust effects or change significantly the mean response amplitude (95.1 ± 32.4% of control, *n* = 5 cells in five larvae). (e) Injection of D2 receptor agonist quinpirole (0.1 mM) significantly decreased evoked responses, with a mean amplitude of 48.7 ± 18.3% of control responses (*n* = 6 cells in six larvae). (f) D2 receptor antagonist raclopride (0.1 mM) significantly increased response amplitude over control values (mean = 149.4 ± 48.8%, *n* = 5 cells in four larvae). MRRN, middle rhombencephalic reticular nucleus. *p < .001 [Color figure can be viewed at http://wileyonlinelibrary.com]

To further characterize the action of DA on olfactomotor transmission, ligands selective for D1 or D2 receptor were pressure‐injected in the medOB (Figure 11). A local microinjection of a D1 receptor agonist, dihydrexidine (0.1 mM), caused a significant decrease of RS cell responses to ON stimulation (Figure 11c). Depression of both amplitude (to 71.5 ± 36.0%; *F* = 11.180, *df* = 2, *p* < .001, *n* = 6 cells in six larvae) and area (to 64.7 ± 87.0%; *χ* ^2^ = 6.222, *df* = 2, *p* = .045, *n* = 6 cells in six larvae) was observed and no significant differences were detected between control and washout of dihydrexidine (amplitude: *t* = 0.329, *p* = .743; area: *q* = 0.943, *p* ≥ .05). However, local microinjection of a D1 receptor antagonist, SCH 23390 (0.5 mM), did not significantly change the amplitude (*F* = 0.263, *df* = 2, *p* = .770, *n* = 5 cells in five larvae) or the area (*F* = 2.269, *df* = 2, *p* = .113, *n* = 5 cells in five larvae) of the RS cell responses (Figure 11d). D2 receptor ligands had more significant effects on RS cell responses. Indeed, a D2 receptor agonist, quinpirole (0.1 mM), induced a marked reduction of RS cell responses (Figure 11e), depressing their amplitude (to 48.7 ± 18.3%; *F* = 43.081, *df* = 2, *p* < .001, *n* = 6 cells in six larvae) and area (to 15.8 ± 45.4%; *χ* ^2^ = 32.889, *df* = 2, *p* < .001, *n* = 6 cells in six larvae). No significant differences were observed between control and washout of quinpirole (amplitude: *t* = 0.672, *p* = .504; area: *q* = 0.667, *p* ≥ .05). Furthermore, a D2 receptor antagonist, raclopride (0.1 mM), had the opposite effect (Figure 11f), increasing both response amplitude (to 149.4 ± 48.8%; *F* = 17.888, *df* = 2, *p* < .001, *n* = 5 cells in four larvae) and area (to 170.4 ± 82.3%; *χ* ^2^ = 8.467, *df* = 2, *p* = .015, *n* = 5 cells in four larvae). No significant differences were observed between control and washout of raclopride (amplitude: *t* = 0.427, *p* = .671; area: *q* = 0.183, *p* ≥ .05). While microinjection of DA receptor agonists (DA, dihydrexidine, and quinpirole) in the medOB reduced the RS cell responses to electrical ON stimulation, raclopride induced an increase of their amplitude. Taken together, these results suggest that DA modulates olfactomotor transmission in the medOB.

## 4 DISCUSSION

**Figure 12:**
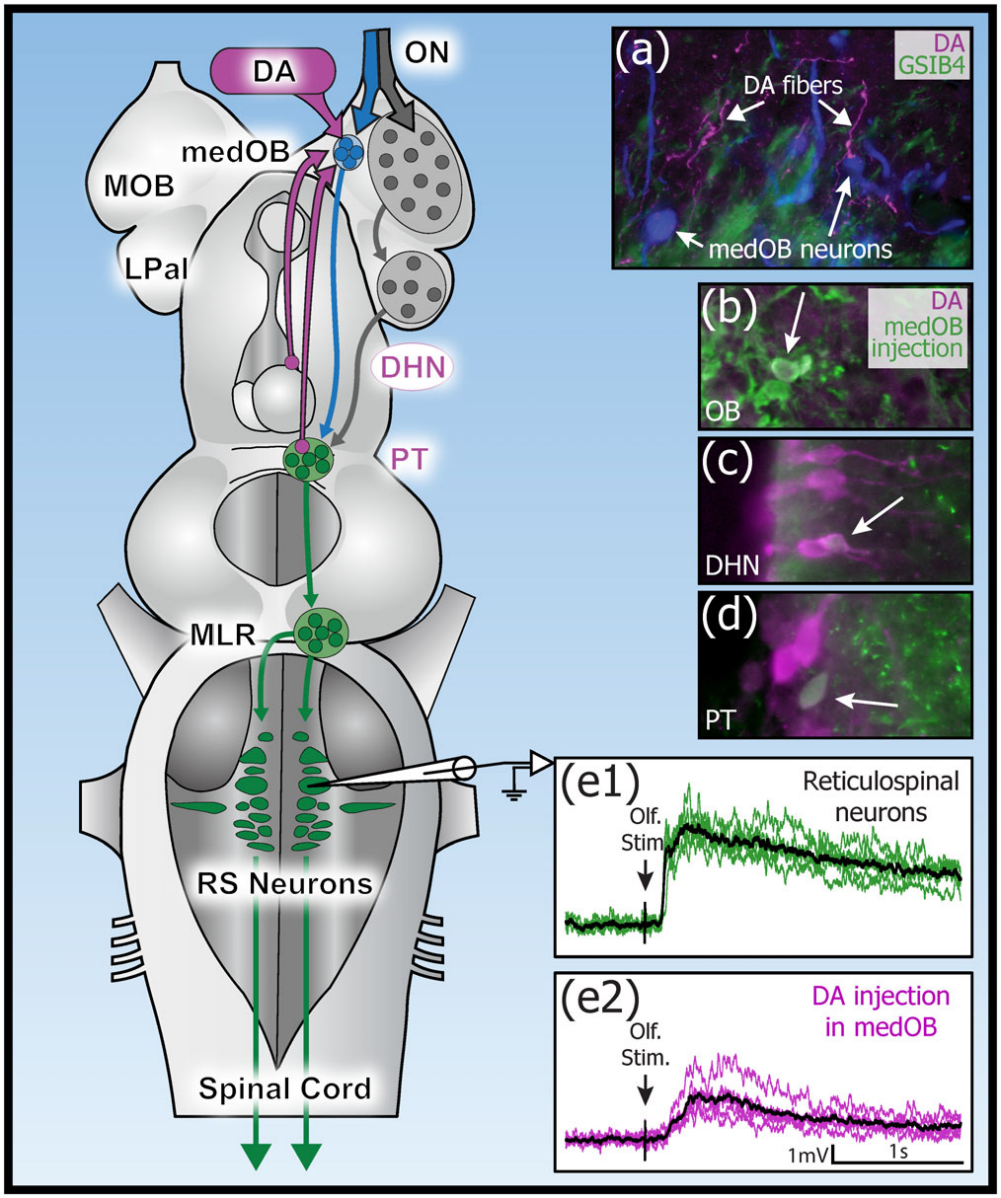
Dopaminergic modulation of the medial olfactory bulb. Schematic representation of the brain illustrating a medial (blue) and lateral (gray) olfactomotor pathway. Both pathways activate the PT which controls downstream locomotor circuitry (green). In the medial pathway, olfactory projection neurons are seen in close proximity to dopaminergic processes (a). The DA+ processes in the medial olfactory bulb (medOB) originated from neurons in the OB (b), DHN (c), and PT (d). The synaptic responses evoked by electrical stimulation of the olfactory nerve in RS cells are decreased following a localized dopaminergic agonist injection in the medOB (e), suggesting a gating of the transmission of olfactory inputs to the motor system in lamprey. DA, dopamine; DHN, dorsal hypothalamic nucleus; LPal, lateral pallium; medOB, medial olfactory bulb; MLR, mesencephalic locomotor region; MOB, main olfactory bulb; ON, olfactory nerve; PT, posterior tuberculum; RS, reticulospinal [Color figure can be viewed at http://wileyonlinelibrary.com]

Results from this study show that DA modulates the transmission of olfactory inputs to brainstem motor centers. Abundant DA+ processes were observed in the medial part of the OB and pharmacological manipulation of DA receptors in this region had physiological effects on olfactomotor activity. Local microinjection of DA agonists (DA, dihydrexidine, or quinpirole) in the medOB decreased responses of RS cells to ON stimulation. Furthermore, microinjection of raclopride in the OB increased these responses, suggesting that D2 receptors are involved in the modulation of olfactory processing. Because DA+ neurons in the OB, DHN and PT were shown to project to the medOB, these different regions might control the activity of the medial olfactomotor pathway through DA transmission (Figure 12).

### 4.1 Dopaminergic processes in the lamprey olfactory bulb

Dopaminergic processes have been observed previously in the lamprey OB, from larval to adult stages, both in the river lamprey (*Lampetra fluviatilis* L.) (Baumgarten, 1972; Pérez‐Fernández et al., 2014; Pierre et al., 1994, 1997; Pierre‐Simons, Reperant, Mahouche, & Ward, 2002; Pombal et al., 1997) and in *P. marinus* (Abalo et al., 2005; Barreiro‐Iglesias et al., 2009, 2010; Fernandez‐Lopez et al., 2017; Yáñez, Molist, Rodríguez‐Moldes, & Anadón, 1992). Compared to previous studies, a major difference in DA immunofluorescence observed in the OB was the presence of two distinct populations of DA+ processes with different developmental patterns and anatomical distributions. The processes from a first type were strongly labeled and denser in the medOB. The processes from a second type contrast sharply: they were weakly labeled and only observed in newly transformed and spawning‐phase adults. They seemed to arise from a local population of similarly labeled DA+ neurons, which are also detected only in adult animals.

Our results suggest that the strongly labeled processes of the first type do not originate from local cell bodies. Although it is possible that such strongly labeled processes arose from weakly labeled DA+ somata in the OB, it is more likely that they stemmed from one of the numerous DA cell groups in the diencephalon (Abalo et al., 2005). In our material, these processes appeared to reach the OB from its caudal aspect passing through the septum (see Figure 2), close to axons from medOB projection neurons exiting the OB (see Figure 4). In larval lampreys, the strongly labeled DA+ processes were detected close to and inside the medOB despite the absence of (or failure to detect) local DA+ neurons in the OB. During prolarval development of *P. marinus*, the earliest detection of DA+ processes reaching the telencephalon coincides with that of DA+ neurons in the PT (Abalo et al., 2005). Most importantly, we provide evidence that DA+ neurons in the PT and DHN project to the medOB. However, PT injections did not allow us to observe anterogradely labeled DA+ processes in the OB, which suggests otherwise. This may be due to a limited number of DA neurons projecting to the medOB from the PT. Although the DA innervation of the OB is traditionally considered to be exclusively local (Smeets & Gonzalez, 2000), extrinsic projections to the OB were notably observed in rats, where a minor portion of SNc neurons send a direct DA projection to the OB (Höglinger et al., 2015). Furthermore, projections from the SNc and the VTA to the OB were observed in the sheep (Lévy, Meurisse, Ferreira, Thibault, & Tillet, 1999) and PT‐OB projections were detected in two species of shark (Yáñez, Folgueira, Köhler, Martínez, & Anadón, 2011). Also, monoaminergic projections modulating OB sensory processing is common in vertebrates, including noradrenergic fibers from the locus coeruleus (Shipley, Halloran, & de la Torre, 1985) and serotoninergic fibers from the raphe nuclei (Broadwell & Jacobowitz, 1976). Hence, in the medOB of lampreys, the DA innervation might originate from extrinsic DA afferents in addition to the intrinsic innervation.

### 4.2 Dopaminergic cell bodies in the lamprey olfactory bulb

Our study shows that DA+ somata were present in the granular layer of the OB in adult lampreys, but were not detected in larvae. The absence of DA+ cells in the OB of larval specimens was also reported previously (Abalo et al., 2005; Pierre‐Simons et al., 2002; Yáñez et al., 1992), but studies on both *P. marinus* and *L. fluviatilis* have described DA+ neurons in adult specimens (Barreiro‐Iglesias et al., 2009; Fernandez‐Lopez et al., 2017; Pierre et al., 1997; Pombal et al., 1997). The phenotype of these cells was confirmed to be DA (TH+/DOPA decarboxylase+/DA+/dopamine β‐hydroxylase‐) in an immunoreactivity study (Pierre et al., 1997). Moreover, the same authors did not find somata containing dopamine‐β‐hydroxylase or phenylethanolamine‐*N*‐methyltransferase in the OB, suggesting the absence of other catecholaminergic (noradrenergic or adrenergic) neurons.

DA+ and TH+ cell groups had the same morphology: in both cases the cell bodies were small, round or ovoid, and often bipolar with processes that arose in opposite directions. Moreover, these two cell groups appear late during development as TH+ cells are undetected in the OB during early larval stages (Pierre‐Simons et al., 2002) and OB DA+ cells are not observed in larval specimens (Abalo et al., 2005; Yáñez et al., 1992; present results). This developmental pattern could be common in vertebrates, as DA cells of the OB, corresponding to the A16 DA cell group (Björklund & Dunnett, 2007), are among the last DA cell groups to be detected during brain development of lampreys (Pierre‐Simons et al., 2002), fishes (Ekström, Honkanen, & Borg, 1992; Manso, Becerra, Molist, Rodriguez‐Moldes, & Anadon, 1993), reptiles (Medina, Puelles, & Smeets, 1994), birds (Puelles & Medina, 1994), mice (Di Porzio, Zuddas, Cosenza‐Murphy, & Barker, 1990), rats (Specht, Pickel, Joh, & Reis, 1981), and humans (Puelles & Verney, 1998). The late development of DA cells in the OB might be conserved in vertebrates and it could explain why DA+ cells are not detected in the lamprey's larval OB.

### 4.3 Dopaminergic modulation of the medial olfactory bulb

A single population of projection neurons located inside the medOB receives sensory inputs exclusively from chemosensory cells in the accessory olfactory organ (Green et al., 2017). The medOB projection neurons then project directly to the PT to drive swimming activity through activation of brainstem RS cells (Daghfous et al., 2018; Derjean et al., 2010). Direct medOB projections to the MLR were also observed (Daghfous et al., 2018). We now show that the injection of DA agonists in the medOB reduces the activation of RS cells in response to ON stimulation. The effects were even more powerful when gabazine, a GABA_A_ receptor antagonist, was injected beforehand in the OB (see Daghfous et al., 2018). The spiking responses in RS cells were then totally suppressed. This suggests that activation of DA receptors in the medOB can lead to a substantially reduced motor output in response to olfactory inputs. This could explain the changes in motor responses to chemical cues that occur during the life cycle of the animal. For example, only during the spawning phase will lampreys respond to migratory pheromones released by larvae (Vrieze & Sørensen, 2001). However, despite dramatic effects in the isolated brain, the effects of DA modulation under more natural conditions are unknown. Future investigations are needed to define how the pharmacological manipulation of DA transmission in the medOB modulates the motor responses to odorants.

Expression of DA receptors was recently characterized in the OB of *P. marinus* and *L. fluviatilis* (Pérez‐Fernández, 2013; Pérez‐Fernández et al., 2014; Pérez‐Fernández, Megias, & Pombal, 2015). D1 or D2 receptors are expressed on somata in the granular layer. In addition, no D4 receptor mRNA‐expressing cells were observed in the OB. Interestingly, cell bodies expressing D2 receptors were also observed in the glomerular layer of the medOB. In our material, microinjection of quinpirole in the medOB decreased RS cell responses to ON stimulation, suggesting that D2 receptor activation plays a role in the inhibitory action of DA on medOB output. Moreover, the D2 receptor antagonist, raclopride, had the opposite effect, suggesting that DA could be released endogenously. Whether DA is released tonically in the medOB or as a feedback mechanism in response to electrical ON stimulation during experimental procedures could not be ascertained in the present study. Additionally, microinjection of the D1 receptor antagonist, SCH 23390, did not significantly change the responses to ON stimulation, although this drug was shown to have physiological effects in the MLR of *P. marinus* (Ryczko et al., 2013). Moreover, D1 receptors have been detected in the OB (Pérez‐Fernández, 2013). Dihydrexidine, a D1 receptor agonist, reduced olfactomotor response amplitude (28.5% decrease), but was less efficient than DA (36.6% decrease) or quinpirole (51.3% decrease), although it was reported that dihydrexidine exhibits more than 10‐fold higher affinity and potency at the D1 receptor than DA (Rosell et al., 2015). Altogether, these physiological results demonstrate that DA exerts a strong modulatory effect on olfactomotor processing in the medOB via D2 and possibly D1 receptors.

Immunofluorescence revealed strongly labeled DA+ processes surrounding the medOB and entering the glomerular neuropil (see Figures 2, 3, 4). However, the cellular targets of DA modulation could not be identified. Dopamine receptors can be expressed on primary olfactory afferents, projection neurons, and/or interneurons as in other vertebrates (Brünig, Sommer, Hatt, & Bormann, 1999; Duchamp‐Viret, Coronas, Delaleu, Moyse, & Duchamp, 1997; Ennis et al., 2001). Based on previous findings and the present results, we hypothesize that projection neurons are the main site of action of DA involved in the modulation of olfactomotor activity.

In rodents, projection neurons express D2 receptors on their dendrites (Gutièrrez‐Mecinas et al., 2005), which are innervated by DA processes (Kasowski, Kim, & Greer, 1999). Moreover, DA reduces the spontaneous and evoked activity of projection neurons (Davila, Blakemore, & Trombley, 2003). In zebrafish, DA has a direct hyperpolarizing effect on projection neurons and modulates their responses to odorants via D2 receptors (Bundschuh, Zhu, Schärer, & Friedrich, 2012). Interestingly, D2 receptor‐expressing cell bodies were detected in the glomerular layer of *L. fluviatilis* (Pérez‐Fernández et al., 2014) and these were exclusively located in the medOB. Although it could be expected that olfactory glomeruli are devoid of cell bodies, the medOB glomerulus is definitely an exception and does contain the somata of PT‐targeting projection neurons in its neuropil (Daghfous et al., 2018; Green et al., 2013; present results). Hence, D2 receptors might be present on the soma or dendrites of medOB projection neurons and directly modulate their activity and output to the PT. Moreover, their activity might also be modulated downstream, as their axons are observed in close proximity to DA+ processes caudal to the medOB (see Figure 4). Indeed, axo‐axonic contacts established by DA fibers have been detected in the striatum of lizards (Henselmans & Wouterlood, 1994) and rats (Bouyer, Park, Joh, & Pickel, 1984; Freund, Powell, & Smith, 1984; Pickel & Chan, 1990), in the median eminence of sheep (Kuljis & Advis, 1989), and the cortex of monkeys (Sesack, Snyder, & Lewis, 1995). Furthermore, in the ventral pallidum of rats, D2 receptors are detected mainly on axons or terminals of non‐DA neurons, suggesting an effect on presynaptic release (Mengual & Pickel, 2002). In the striatum, the activity of cortical projections is presynaptically modulated via D2 receptors (Garcia‐Munoz, Young, & Groves, 1991; Schwarcz, Creese, Coyle, & Snyder, 1978). As DA neurons establish axo‐axonic contacts, DA+ processes close to medOB projection axons may have modulatory effects on olfactomotor transmission through axo‐axonic synapses.

Because somata expressing the D2 receptor are observed in the medOB in *L. fluviatilis* (Pérez‐Fernández et al., 2014) and our electrophysiological data suggest an inhibition of the olfactomotor processing via D2 receptors in the medOB, we hypothesize that DA processes innervating the medOB act mainly on projection neurons via D2 receptors to regulate odor‐driven locomotion. Future studies are needed to define the exact localization of DA receptors in the medOB.

### 4.4 Behavioral consequences

Lampreys rely extensively on olfaction to regulate their behavior. Furthermore, olfactory‐evoked behaviors vary across life and are adapted to the developmental stage of the animal. For example, upon reaching the reproductive stage, lampreys stop feeding and are attracted to migratory pheromones (Sørensen et al., 2005; Vrieze, Bergstedt, & Sørensen, 2011). It has been suggested that the medial olfactomotor pathway is wired to quickly generate locomotion upon detection of numerous odorants (Derjean et al., 2010). A modulation of this pathway would thus allow for a wide variety of behavioral responses. Moreover, the olfactory sensory input originating from the accessory olfactory organ requires only two synapses to activate the motor systems. A modulation at the level of the OB would be an efficient way to regulate motor responses at the first relay of this pathway.

The evidence collected here suggests that DA modulation of the medOB exists from the larval to the spawning phase. Indeed, strongly labeled DA+ processes were present with a constant pattern of innervation of the medOB in larval, newly transformed adult and spawning‐phase adult lampreys. These processes could produce a physiological effect before the development—or detection—of DA+ somata in the OB and maintain their function after metamorphosis as DA microinjection in the larval medOB produced effects on olfactomotor transmission that persisted in the postmetamorphic stage. Dopaminergic modulation in the olfactomotor pathway could thus gate motor responses that would be inappropriate in relation to the life stage of the animal.

Another effect of DA may be the fine‐tuning of medOB activity in response to odorants. The activation of the medial olfactomotor pathway could produce appetitive goal‐directed locomotion, as the medOB is activated by amino acids, bile acids, and pheromones (Green et al., 2017), all of which can elicit goal‐directed swimming (Bjerselius et al., 2000; Johnson, Yun, Thompson, Brant, & Li, 2009; Kleerekoper & Mogensen, 1963; Li et al., 2002). In the natural environment, these chemical cues are encountered in a wide range of concentrations and DA modulation in the medOB might contribute to olfactory processing during tracking behaviors. In rodents, DA modulation of glomerular activation was proposed to increase the range of odorant levels processed by the OB (Ennis, Hamilton, & Hayar, 2007). Dopaminergic inhibition in the medOB could thus dynamically adapt olfactory sensitivity, which would allow the animal to follow an olfactory target more efficiently. Such mechanism may increase the range of odorant concentrations inducing an appropriate locomotor response, which is to swim toward their source.

In addition to a local DA source of inputs in the medOB, the two diencephalic DA cell populations projecting to the medOB identified here could provide means for adapting olfactomotor behaviors in different contexts. First, one source of DA innervation originates from the PT, a DA nucleus homologous to the SNc/VTA (Baumgarten, 1972; Pombal et al., 1997). As medOB projection neurons reach directly the PT to drive locomotion (Daghfous et al., 2018; Derjean et al., 2010), there are reciprocal connections between the medOB and the PT, which would thus allow PT neurons to control the inputs they receive. Interestingly, a reciprocal connection also exists between the PT and the tectum (Pérez‐Fernández et al., 2014). It was recently found that DA neurons in the PT are activated by visual stimuli, coding saliency (Pérez‐Fernández, Kardamakis, Suzuki, Robertson, & Grillner, 2017). In this study, authors reported that the PT modulates visuomotor transformations mediated in the tectum by modifying tectal neuron responsiveness to visual stimuli via direct DA projections. Similarly, the DA projections of the PT to the medOB could modulate olfactory processing so that odorants generate a motor output less effectively. This mechanism would allow for flexibility in the motor output evoked by the medial olfactomotor pathway. Additionally, the DHN contained CSF‐contacting DA+ cell bodies projecting to the medOB. Hypothalamic projections to the olfactory system are common in vertebrates and exert modulatory effects to control odor‐driven behaviors (Gascuel et al., 2012). In lamprey, TH+ cells of the DHN are in contact with the CSF and give rise to long extrahypothalamic pathways reaching telencephalic structures (Pierre et al., 1994). Therefore, the CSF‐contacting neurons may modulate the activity of the medOB to adjust the behavioral output according to the functional state of the hypothalamus or according to the rate of diverse hormones or other chemical substances in the CSF.

## 5 CONCLUSIONS

In mammals, DA transmission in the OB is important for odor discrimination (Pavlis et al., 2006; Tillerson et al., 2006; Wei et al., 2006) and learning (Escanilla et al., 2009) and DA interneurons are produced throughout life to maintain these functions. In lampreys, the role of DA modulation appears less complex with a more direct impact on motor output. We show here the presence of a powerful modulatory effect of DA on lamprey RS cell responses to olfactory inputs. The DA innervation is not only intrinsic but also originates from sources outside of the OB. Altogether, our results provide new insights into the control of a neural circuit transforming an olfactory input into a motor output in lampreys. Future directions could focus on the impact of DA transmission on motor behaviors that are induced by the direct application of odorants on olfactory sensory neurons.
